# The practical operation and consequences of glucose measurement by pilots with diabetes

**DOI:** 10.1111/dme.15472

**Published:** 2024-11-09

**Authors:** Ka Siu Fan, Antonios Manoli, Fariba Shojaee‐Moradie, Ewan Hutchison, Felice Strollo, Gerd Koehler, Julia K. Mader, David Russell‐Jones, EASA Diabetes Consortium

**Affiliations:** ^1^ CEDAR Centre Royal Surrey County Hospital Guildford UK; ^2^ Department of Nutritional Sciences University of Surrey Guildford UK; ^3^ Division of Endocrinology and Diabetology, Department of Internal Medicine Medical University of Graz Graz Austria; ^4^ Civil Aviation Authority Crawley UK; ^5^ Endocrinology and Diabetes Unit IRCCS San Raffaele Pisana Rome Italy; ^6^ Aeromedical Section Vienna Austria

**Keywords:** aviation, blood glucose, continuous glucose monitoring (CGM), pilot, self‐monitoring of blood glucose (SMBG)

## Abstract

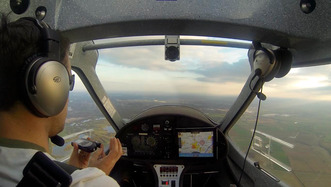

Managing health conditions in the aviation industry is crucial to the safe and effective operation of aircraft. With diabetes on the rise, its safety and health implications should be considered in pilots and air traffic control officers (ATCOs). The incapacitation risks of hypoglycaemia remain a leading concern for regulators, and where people with diabetes are allowed to operate, medical fitness and frequent glucose monitoring must be demonstrated.^1^

**TABLE 1:** Summary of participants' attitude and practical operations of using CGM and SMBG.

	CGM	SMBG
Respondent attitudes and ratings of the glucose measurement method (n/N)		
Very satisfied or satisfied with the attention required to take a measurement	6/6	3/6
Very satisfied or satisfied with convenience of the measurement method	6/6	3/6
Very satisfied overall with the modality	6/6	2/6
Very confident in the results from the modality	6/6	4/6
Very confident in acting on the readings	6/6	5/6
Time required to take measurement		
Min Time	1 S	4 S
Max Time	20 S	120 S
Mean	6.1 S	46.9 S
Median	4.5 S	41 S

Abbreviations: CGM, continuous glucose monitoring; SMBG, self‐monitoring blood glucose.

Self‐Monitoring of Blood Glucose (SMBG) capillary glucose measurements is typically accepted in aviation.^1^ Continuous Glucose Monitoring (CGM) systems provide near‐instant measurements of interstitial glucose levels via disposable skin sensors. As they are convenient and demonstrate comparable mean absolute relative difference to SMBG, people with diabetes generally prefer CGM.^2^, ^3^ Pilots and ATCOs can benefit from the tighter glucose management associated with CGM to meet medical certification and on‐duty needs without additional operational load. This study investigated the perception of pilots with diabetes on the CGM and SMBG.

This project was conducted in conjunction with the Safe Use of New technologies in Diabetes in Flight (SUNDIF) projects funded by the European Union Aviation Safety Agency. This involved studying type 1 diabetes, insulin pumps, and closed‐loop systems by simulating aviation‐related atmospheric pressure changes in a hypobaric chamber.^4^, ^5^ Pilots with diabetes may be certified to fly under the ARA.MED.330 Protocol of the UK Civil Aviation Authority, and were recruited for SUNDIF.^6^, ^7^ Participants were surveyed for their satisfaction and confidence in using SMBG and CGM. The steps required to take glucose measurements in the hypobaric chamber, including preparation of glucometer and strip, were timed by the authors with triplicate repeats.

All six pilots, with a median age of 40 (ranged 20–61) years, responded in full. All participants were male, had type 1 diabetes, and used either Medtronic Guardian 4 (3/6) or Dexcom G6 (3/6). Participants' insulin pumps included: Medtronic 780G (3/6), Tandem t:slim X2 (1/6), Omnipod DASH (1/6), and mylife YpsoPump (1/6).

The mean time required to measure glucose using CGM and SMBG were 6 (± 4.8) and 47 (± 28) s, respectively. The slowest CGM measurement was 20 s, compared to 120 s for SMBG. Across a standard 5‐h flight, pilots under the ARA.MED.330 Protocol would measure their glucose ≥7 times. Extrapolation using our measurements suggests a potential saving of 286 s (42.7 vs. 328) throughout the flight.

All users preferred CGM over SMBG (Table 1). All users reported being very‐satisfied with CGM (6/6) whereas only half were satisfied or very‐satisfied with SMBG (3/6). Few pilots were satisfied or very‐satisfied with the attention (3/6) and convenience (2/6) required by SMBG. Pilots were very confident in acting on CGM results (6/6 vs. 4/6) and trusted CGM a lot compared to SMBG (6/6 vs. 5/6). All respondents valued CGM trend arrows, trend graphs, and glucose alerts.

Only a few aviation protocols currently allow insulin‐dependent pilots to fly.^1^ These all stipulate detailed clinical evaluations, including blood tests, complication screening, and hypoglycaemic awareness assessments. The most important consideration is the in‐flight glucose monitoring and avoidance of incapacitation.

In comparison with SMBG, CGMs can be effective at improving HbA1c and reducing severe hypoglycaemia episodes in type 1 diabetes.^8^ Together, these attributes provide pilots the means to operate aircraft within stricter glucose ranges and prevent incapacitation. This was reflected in the overall trust and confidence in using CGMs in our findings and suggests that pilots view CGM as at least equivalent to SMBG, especially given the encouraging preliminary evidence in aviation.^6^, ^9^

Our recent evidence evaluating the safety of insulin‐treated pilots has been encouraging. With our 7‐year observational study demonstrating no significant concerns in insulin‐treated pilots, the focus then extends to glucose measurement methods.^5^ Our feasibility study compared pilots' in‐flight CGM and SMBG readings in ARA.MED.330 protocol and also demonstrated a strong correlation (*r* = 0.843; *p* < 0.001) to support its accuracy.^6^ Additionally, our preliminary hypobaric study on the closed‐loop systems also demonstrated no significant concerns with the use of CGM.^4^

Perception aside, the practicalities of measurements also differed: SMBG were significantly slower to perform, was less convenient, and required more attention. Therefore, the time saved by using CGM can be significant over time. However, the additional application and “warm up” time for CGMs, and its approximately weekly replacements, may also factor into pilots' views.^10^

This study was limited by its size and the unvalidated survey. Our findings might not represent all pilots or ATCOs, even if they work under similar circumstances. Our measurements were conducted in an environment similar to the real‐world to approximate the in‐flight use and impact of CGMs. Regardless, CGMs hold the potential to improve diabetes management within aviation. Although CGMs may need calibration and frequent replacements, the convenience and accuracy of CGM systems are perceived by pilots to be comparable, if not better, than SMBG. Given the time‐pressured environment in aviation, its implementation may be beneficial to both pilots and ATCOs.

## FUNDING INFORMATION

This project was conducted in conjunction with the Safe Use of New technologies in Diabetes in Flight (SUNDIF; NCT06408558) study funded by the EASA Horizon work programme 2021–2022 (IRAS 31859; protocol number FHMS 2022 15). Device manufacturers were not involved in the study design or study protocol. No other source of funding was received for this study.

## CONFLICT OF INTEREST STATEMENT

JKM is a member of advisory boards of Abbott Diabetes Care, Becton‐Dickinson, Boehringer Ingelheim, Eli Lilly, Embecta, Medtronic, NovoNordisk A/S, Roche Diabetes Care, Sanofi‐Aventis, Viatris and received speaker honoraria from A. Menarini Diagnostics, Abbott Diabetes Care, AstraZeneca, Boehringer Ingelheim, Dexcom, Eli Lilly, Medtrust, MSD, NovoNordisk A/S, Roche Diabetes Care, Sanofi, Servier, Viatris and Ypsomed. She is shareholder of decide Clinical Software GmbH and elyte Diagnostics and serves as CMO of elyte Diagnostics. DRJ receives research funding or advisory board honraria from Abbott Diabetes Care, Dexcom, Astra Zeneca, Eli Lilly, Medtronic, Novartis, Novo Nordisk A/S and Sanofi‐Aventis. GK is contracted as an independent advisor to Austro Control and has received research funding, speaker and advisory board honoraria from Astra Zeneca, Amgen, Boehringer‐Ingelheim, Daiichi Sankyo, Eli Lilly, Novartis, Novo Nordisk, Roche Diagnostics and Sanofi. All other authors report no potential conflicts of interest.
